# Identification and characterization of novel compound heterozygous variants in *FSHR* causing primary ovarian insufficiency with resistant ovary syndrome

**DOI:** 10.3389/fendo.2022.1013894

**Published:** 2023-01-10

**Authors:** Xiaopan Chen, Linjie Chen, Yang Wang, Chongyi Shu, Yier Zhou, Ruifang Wu, Bihui Jin, Leixiang Yang, Junhui Sun, Ming Qi, Jing Shu

**Affiliations:** ^1^ Reproductive Medicine Center, Department of Reproductive Endocrinology, Zhejiang Provincial People’s Hospital, Affiliated People’s Hospital, Hangzhou Medical College, Hangzhou, China; ^2^ Department of Genetic and Genomic Medicine, Zhejiang Provincial People’s Hospital, Affiliated People’s Hospital, Hangzhou Medical College, Hangzhou, China; ^3^ School of Laboratory Medicine and Bioengineering, Hangzhou Medical College, Hangzhou, China; ^4^ The Second Clinical Medical School of Wenzhou Medical University, Wenzhou, China; ^5^ Reproductive Medicine Center, The First Affiliated Hospital of Wenzhou Medical University, Wenzhou, China; ^6^ Department of Cell Biology and Medical Genetics, School of Medicine, Zhejiang University, Hangzhou, Zhejiang, China

**Keywords:** primary ovarian insufficiency, resistant ovary syndrome, follicle-stimulating hormone receptor, compound heterozygous variant, transmembrane helix

## Abstract

Primary ovarian insufficiency (POI) is among the foremost causes of women infertility due to premature partial or total loss of ovarian function. Resistant ovary syndrome (ROS) is a subtype of POI manifested as normal ovarian reserve but insensitive to gonadotropin stimulation. Inactivating variants of follicle-stimulating hormone receptor (FSHR), a class A G-protein coupled receptor, have been associated with POI and are inherited *via* an autosomal recessive pattern. In this study, we investigated the genetic causes of a primary infertility patient manifested as POI with ROS, and elucidated the structural and functional impact of variants of uncertain significance. Next-generation sequencing (NGS) combined with Sanger sequencing revealed novel compound heterozygous *FSHR* variants: c.1384G>C/p.Ala462Pro and c.1862C>T/p.Ala621Val, inherited from her father and mother, respectively. The two altered amino acid sequences, localized in the third and seventh transmembrane helix of FSHR, were predicted as deleterious by *in silico* prediction. *In vitro* experiments revealed that the p.Ala462Pro variant resulted in barely detectable levels of intracellular signaling both in cAMP-dependent CRE-reporter activity and ERK activation and displayed a severely reduced plasma membrane receptor expression. In contrast, the p.Ala621Val variant resulted in partial loss of receptor activation without disruption of cell surface expression. In conclusion, two unreported inactivating *FSHR* variants potentially responsible for POI with ROS were first identified. This study expands the current phenotypic and genotypic spectrum of POI.

## Introduction

Primary ovarian insufficiency (POI) is an etiologically and clinically heterogeneous condition caused by the early loss or complete absence of ovarian activity (1, 2). POI affects roughly 1%–2% of females <40 years old (3), making it among the foremost causes of women infertility. Resistant ovary syndrome (ROS) is a special form of POI characterized by the presence of normal ovarian reserve and resistance to gonadotropin (GN) stimulation (4–6). The etiopathogenesis of POI is multifactorial, including genetic alterations, autoimmune and metabolic disorders, viral infections, and environmental or iatrogenic factors (7). Genetic factors represent the most commonly identified causes; however, the current understanding of its hereditary basis is incomplete (8, 9).

The follicle-stimulating hormone receptor (FSHR) is expressed in granulosa cells from the primary follicle stage onwards (10, 11) and rigidly controls follicle development in response to cyclic pituitary FSH discharge (11, 12). FSHR belongs to a highly conserved subfamily of G protein-coupled receptors with a remarkably long amino-terminal extracellular domain (ECD), an intracellular carboxyl-terminal tail (C-tail), and a typical structural architecture comprising seven transmembrane helixes (TMH) interconnected by three extracellular loops (ECL) and three intracellular loops (ICL) (13, 14). Upon FSH binding, the receptor adopts a conformational change and subsequently couples to Gαs protein, which in turn activates the adenylyl cyclase, resulting in increased cAMP signaling (15, 16). Variations in any of these domains by alterations in primary DNA sequences may potentially lead to receptor dysfunction and eventually to disease (17).

Genetic variants that inactivate protein-coding genes, collectively known as loss-of-function variants, are often single-nucleotide variants that disrupt the structure and function of the protein. *FSHR* inactivating variants, one of the rare causes of POI, are inherited recessively and require homozygous or compound heterozygous status to develop the clinical phenotype (18). After the first POI-causative FSHR variant-p.Ala189Val in the ECD was reported (19), diverse inactivating variants have been identified in different FSHR domains, including the ECD (20–29); the second, fourth, and sixth TMH (26, 29–34); the first, second, and third ECL (22, 28, 35, 36); second and third ICL (21, 37); and the C-tail (27). However, pathogenic inactivating variants in the third and seventh TMH of FSHR have not been reported so far in patients with POI.

Numerous evidence indicates that the clinical manifestations of *FSHR* variants are not uniform, ranging from primary amenorrhea with puberty disorders to secondary amenorrhea, oligomenorrhea, and premature menopause, depending on the specific domain and type of variant and the degree of inactivation. Thus, expanding the POI-causing variant spectrum of *FSHR* is crucial to understand the etiopathogenesis of POI and for better clinical diagnosis that involves patients and their offspring. Herein, we reported two novel *FSHR* variants (c.1862C>T/p.Ala621Val and c.1384G>C/p.Ala462Pro) in a compound heterozygous state in a Chinese infertile woman who manifests hypergonadotropic hypogonadism (HH) with oligomenorrhea, presence of normal ovarian reserve, and resistance to exogenous GNs: three clinical characteristics of POI with ROS. Using *in vitro* functional experiments, we characterized the molecular features underlying this disease phenotype, thereby providing new insights into POI with ROS. To our knowledge, this is the first study identifying pathogenic variants in the TMH3 and TMH7 of FSHR as causative variants for POI.

## Materials and methods

### Ethical compliance and informed consent

The study procedures were reviewed and approved by the Institutional Ethics Committee of Zhejiang provincial People’s Hospital (Approval number: 2019KY205). Written informed consent was obtained from all participants. All genetic materials were handled in accordance with the National Regulation on Human Genetic Resources.

### Case presentation and medical history

A 29-year-old Chinese female was referred to our outpatient center for 5 years of primary infertility. The female patient reached menarche at age 15 and had experienced irregular menstrual cycles (15 - 180 days) in a progressively prolonged manner. At the age of 21 years, she consulted a local hospital for irregular periods, and after sex hormonal evaluation, she was diagnosed with ovarian dysfunction and began to intermittently take exogenous hormone replacement therapy to induce artificial menstrual cycles. After the age of 21 years, her menstrual cycle never appeared again without hormone therapy. A dominant follicle was detected during an occasional ultrasound examination when she was 26-year-old. She was otherwise healthy and had an unexceptional past clinical history. No family history of consanguinity, reproductive anomaly or infertility was documented.

### Fertility investigations and ROS diagnosis

Upon examination, the patient’s height and weight were found to be 160 cm and 62.5 kg, respectively. Physical examination of secondary sex characteristics revealed normal breast development, normal appearance of female external genitalia, and normal armpit and pubic hair. Hormonal assays were carried out in the presence of low doses of estrogen (Estradiol Valerate Tablets 1-2mg/d, Delpharm Lille S.A.S., Lys-lez-Lannoy, France). The repeated basal hormonal evaluations in our department revealed elevated serum FSH (35.98 and 37.73 IU/L) and luteinizing hormone (LH) (21.23 and 21 IU/L) (**Table 1**). The patient’s thyroid stimulating hormone (TSH) level was normal (2.34 mIU/L), as was her prolactin (PRL) (10.03 ng/mL) (**Table 1**). Despite the increase in GNs, the patient’s anti-Müllerian hormone (AMH) level was 2.89 ng/mL (**Table 1**), suggesting a normal ovarian reserve. Transvaginal ultrasonography examination displayed a normal-sized uterus and normal-sized bilateral ovaries, consisting of 3–5 small antral follicles 3–5 mm in diameter (**Supplemental Figure**). The patient had no autoimmune condition, including absence of anti-nuclear and anti-cardiolipin antibodies. Immunoglobulin and natural killer cell levels were within normal ranges. Chromosome analysis and Fragile X DNA test were normal. No other relevant clinical features were observed.

**Table 1:** Clinical characteristics of the proband.

Age at presentation (years)	Age at menarche (years)	Second sex characteristics	Ovary size	Follicle count a	Follicle size (mm) a	FSH (IU/L) a	LH (IU/L) a	E2 (pg/ml) a	PRL (ng/ml) a	TSH (mIU/L) a	AMH (ng/m 1)	Autoimmune screening	Karyotype and Fragile X screening
29	15	Normal	Normal	3-5 each	Up to 5	35.98 and 37.73b	21.23 and 21.0b	45.0 and 41.9b	10.03	2.34	2.89	Normal	Normal

a, Measurements were conducted at follicle stage; b, Repeated test with 4 weeks apart; Reference values for follicle phase: FSH: 3.03-8.08 IU/L; LH: 1.80-11.78 IU/L; E: 21.00-251.00 pg/ml; PRL: 5.18-26.53 ng/ml; TSH: 0.4-4 mIU/L.

After failing to observe follicle development in the natural cycle, an initial attempt of controlled ovarian stimulation (COS) was performed by a daily combination of 50 mg clomiphene and 150 IU human menopausal gonadotropins (hMG). However, the stimulation was cancelled after 8 days of treatment with a total dose of 1,200 IU FSH, as no ovarian follicle development was observed (peak serum estradiol level, 39 pg/mL). To exclude the possibility that the ovarian unresponsiveness to exogenous GNs was due to stimulation insufficiency, another COS cycle with increased daily hMG dose and prolonged stimulation was conducted. Despite increasing the daily dose of exogenous GNs up to 375 IU per day, growth of immature follicles remained unresponsive even after 15 days of stimulation with total FSH dose of 4,350 IU. Thus, the patient was clinically diagnosed with ROS according to previously described criteria (6, 38).

### Endocrine assays and follicular monitoring

The FSH, LH, estrogen (E_2_), PRL, and TSH concentrations were tested by a set of commercial enzyme immunoassay kits (7K72-78 & 2P13-40, Abbott, Chicago, USA). The ovarian reserve was measured by an AMH detection kit (C86002, YHLO, Shenzhen, China). Monitoring of follicle growth and development was conducted by transvaginal ultrasonography (Hitachi, Tokyo, Japan).

### Massively parallel sequencing

Whole-exome sequencing was conducted on DNA samples isolated from the patient’s peripheral blood leukocytes, according to the manufacturer’s manual (Agilent Technologies, Santa Clara, USA; Illumina, San Diego, USA). In brief, genomic DNA was fragmented, barcoded, purified, and hybridized with capture probes during library preparation for massively parallel sequencing. The captured sequences were then enriched with streptavidin-conjugated paramagnetic beads and further amplified before being subjected to NGS. Clusters were generated and sequenced on the Illumina NextSeq 550 system using the Illumina Nextseq High Output Kit (20024908) to obtain approximately 90 million reads per sample (2 × 151 cycles). After quality assessment, the resulting paired-end reads were mapped to the GRCh37 assembly (hg19) of the human genome. All called variants were annotated based on public databases, including the 1KGB (39), ExAC (39), gnomAD (40), dbSNP (41), OMIM (42), ClinVar (43), and LOVD (44). Major focus of the analysis was on the 4 relevant disease-causing genes (*FSHB, FSHR, LHB and LHCGR*). Variant interpretation was based on the gene clinical and pathogenic relevance according to ACMG guidelines (45).

### Sanger sequencing

Validation of NGS results was conducted by Sanger sequencing. The primers used to amplify target region of the human *FSHR* gene were designed as follows: 5′-CAAACTGGGGCAGGCTGTGATG-3′ and 5′-CTTGCATTTCATAGCAGCCACAC-3′. All the PCRs were carried out at 50 μl reaction volume using 100 ng genomic DNA on an ABI GeneAmp 9700 PCR system (Applied Biosystems, Bedford, USA), and the amplified fragments were subsequently extracted from the agarose gel, purified, and directly sequenced by Big Dye Direct Cycle Sequencing (4458688, ThermoFisher Scientific, Waltham, USA) on an ABI 3730 Genetic Analyzer (Applied Biosystems, Bedford, USA). The nucleotide sequences were blasted with the published sequence of human *FSHR* gene (http://www.ncbi.nlm.nih.gov).

### Protein topology and function prediction

Protein topology prediction of *FSHR* variants was conducted using the TMHMM architecture (46). Variant pathogenicity prediction was performed using three online tools: PolyPhen-2 (47), SIFT (48), and MutationTaster (49). Evolutionary conservation analysis was performed based on multiple-alignment of the amino acids across multispecies from the HomoloGene database.

### Construction of plasmid vectors

The wild-type (WT) FSHR plasmid was constructed by subcloning human FSHR cDNA from pCR-BluntII-TOPO-FSHR (P19532, Miaolingbio, Wuhan, China). The obtained polymerase chain reaction products were then cloned into pcDNA3.1 and pEGFP-N1 with the Seamless Cloning Kit (D7010, Beyotime, Shanghai, China). Point mutations were performed by using the QuickMutation Kit (Beyotime, Shanghai, China). The sequences of primers used for plasmid construction were provided in **Supplemental Table**. The pCRE-luciferase vector was kindly provided by Dr. Naiming Zhou (50). All vectors were thoroughly sequenced to confirm sequence integrity.

### Cell maintenance and transfection

The HEK293 cell line was routinely cultured in Dulbecco’s modified Eagle’s medium (Gibco, Waltham, USA) containing 10% heat-inactivated fetal bovine serum (SV30087, Hyclone, Logan, China) as described previously (51). Transfection of plasmid constructs into cells was conducted using lipofectamine 3000 reagent (L3000-015, Invitrogen, Carlsbad, USA). Variants were transfected alone (3 μg) to mimic the homozygous state, or together in equimolar concentration (1.5 μg each) to mimic the compound heterozygous state.

### cAMP-dependent CRE-reporter assay

Cells pre-seeded in a 96-well plate were co-transfected with either an expression vector (pWT-cDNA or variants) and reporter vector (pCRE-Luc). At 48 h after transfection, cells were incubated with the DMEM containing various concentrations of recombinant FSH (S20150007, Jinsai, Changchun, China) for 4 h at 37°C. cAMP-dependent CRE-luciferase activity was measured using the firefly luciferase assay system (RG042S, Beyotime, Shanghai, China) with a multiple function microplate reader (Tecan, Männedorf, Switzerland). Briefly, the culture medium in the plate was removed, and 50 μL per well reporter lysis buffer was added immediately. The plate was then placed on a horizontal shaker for complete lysis. The cell lysate was collected and centrifuged in 10,000 × g for 5 min. Then, 20 μl supernatant was mixed with 100 μl luciferase substrate and incubated for 5 min. Finally, the mixture was transferred into a 96-well plate, and luminance was read on Tecan luminometer 1 s/per well at the 470 nM. Efficacy and potency were determined by a three-parameter nonlinear logistic regression using GraphPad Prism 7.0 (Graph Pad Software, San Diego, USA).

### Western blot analysis of ERK activation

Cells transiently expressing wild-type receptor or variants were stimulated with the FSHR agonist (S20150007, Jinsai, Changchun, China) for the indicated durations. Drug incubation was terminated by washing the cells with ice-cold phosphate buffered saline followed by the addition of lysis buffer containing complete protease inhibitor (P1010, Beyotime, Shanghai, China) and phosphatase inhibitors cocktail (P1082, Beyotime, Shanghai, China). Equal protein amounts from cell lysates were electrophoresed on a 10% SDS-polyacrylamide gel, and then transferred to a PVDF membrane and incubated with rabbit monoclonal anti-phospho-ERK1/2 antibody (4370, Cell Signaling, Danvers, USA) followed by stripping and reprobing with anti-total ERK antibody (4695, Cell Signaling, Danvers, USA) according to manufacturers’ protocols. Chemiluminescence was detected using an ECL substrate (FD8000, Fdbio science, Hangzhou, China) with ChemiDoc Touch Imaging System (Bio-Rad, Hercules). All immunoblots were semi-quantified using the Adobe Photoshop CC software, and ERK1/2 activation was calculated as the level of phosphor-ERK1/2 normalized by the total-ERK.

### Cell-surface receptor expression

Cells pre-seeded in a coverslip-covered 12-well plate were transiently transfected with pWT-EGFP, pA462P-EGFP, or pA621V-EGFP. At 48 h after transfection, the coverslip seeded with transfected cells was invertedly placed on a microscope slide and observed immediately under a confocal laser-scanning microscope (Leica, Wetzlar, Germany). The transfection efficiency was determined by counting number of cells that express fluorescence divided by the total number of observed cells. Quantification of the FSHR level on cell membrane by their mean fluorescent signal intensity was measured using ImageJ (NIH, Bethesda, USA); moreover, the plugin of MorphoLibJ in ImageJ was used to create segmentation borders. Furthermore, a region of interest area was created for a final measure.

### Statistical analyses

Statistical analysis of *in vitro* experimentation was performed with GraphPad Prism software using data derived from ≥3 different biological replicates. Collected data were examined for normality of distribution using Shapiro–Wilk normality test. Comparisons between groups were carried out using one-way analysis of variance followed by Tukey’s multiple comparisons tests for *post-hoc* analysis. Differences between groups were considered statistically significant at *P* < 0.05.

## Results

### Genetic analysis and functional prediction of *FSHR* variants

**Figure 1:**
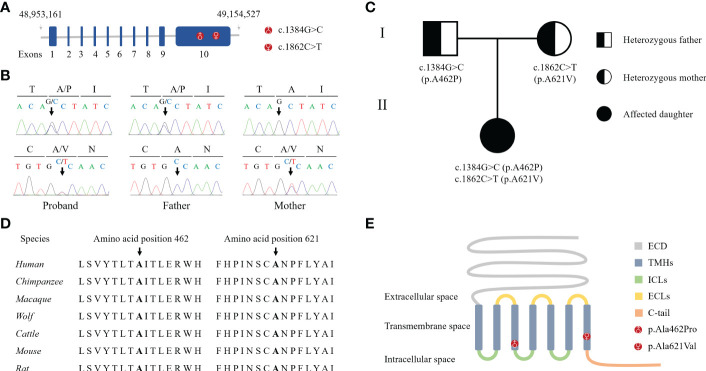
Identification of novel compound heterozygous variants in *FSHR*. **(A)** Schematic representation of the *hFSHR* gene and localization of newly identified variants. Size of exons is drawn to scale. **(B)** Validation of NGS by Sanger sequencing. **(C)** Pedigree of the family examined in the present study. **(D)** Multiple amino acid sequences alignment of FSHR target sites and their two flanks across different species. **(E)** Schematic representation of *hFSHR* and localization of newly identified variants.

Bioinformatics analysis following NGS revealed two missense variants. The first variant was a transition of guanine to cytosine at nucleotide position 1384 in Exon 10 producing an Alanine to Proline replacement at position 462 (*FSHR*_ex10 c.1384G>C/p.Ala462Pro). The second variant was a transition of cytosine to thymine at nucleotide position 1862 in Exon 10 of *FSHR* gene resulting in an amino acid substitution of Alanine to Valine at position 621 (*FSHR*_ex10 c.1862C>T/p.Ala621Val). Sanger sequencing confirmed the suspected variants and showed that the patient’s parents were heterozygous carriers; the father and mother being a heterozygous carrier of c.1384G>C/p.Ala462Pro and c.1862C>T/p.Ala621Val variants, respectively (**Figures 1A-C**). Both variants were neither found in public population databases (1KGB, ExAC, gnomAD, and dbSNP) nor in disease databases (OMIM, ClinVar, and LOVD) (**Table 2**). Multiple sequence alignments were performed for FSHR proteins form different species; both A462 and A621 were highly evolutionarily conserved over multiple species (**Figure 1D** and **Table 2**). Protein topology prediction of the variants revealed that p.Ala462Pro and p.Ala621Val were localized in the third and seventh TMH of FSHR, respectively (**Figure 1E**). Furthermore, the two variants were predicted to be “Probably damaging” by PolyPhen-2, “Disease causing” by MutationTaster, and “Intolerated” by SIFT, indicating potential pathogenic effects of these two variants (**Table 2**). Thus, both p.Ala621Val and p.Ala462Pro were ranked as variants of uncertain significance according to ACMG pathogenicity assessing criteria (**Table 2**).

**Table 2:** Bioinformatic prediction of the *FSHR* variants.

Variant	1KGB	ExAC	gnomAD	dbSNP	ClinVar	OMIM	LOVD	PolyPhen-2	SIFT	Mutation Taster	Conservation	ACMG classification
c.1384G>C (p.Ala462Pro)	N/F	N/F	N/F	N/F	N/F	N/F	N/F	D/C	D/C	D/C	H/C	VUS
c.1862C>T (p.Ala621Val)	N/F	N/F	N/F	N/F	N/F	N/F	N/F	D/C	D/C	D/C	H/C	VUS

N/F, Not found; D/C, Disease causing; H/C, Highly conserved; VUS, Variants of uncertain significance.

### A462P and A621V variants were significantly impaired in cAMP-dependent CRE-luciferase activity

**Figure 2:**
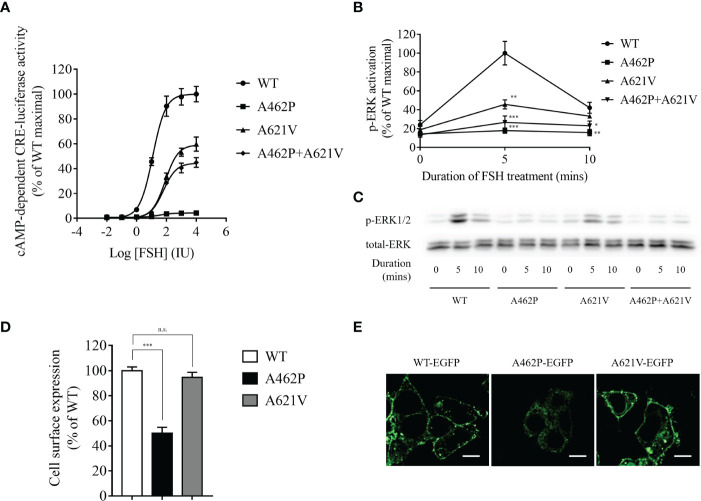
Functional evaluation of variants. **(A)** Dose-dependent curve of cAMP-dependent CRE-luciferase activity for *FSHR* variants upon FSH treatment. Variant transfected HEK293 cells were incubated with various FSH concentrations for 4 h. Data are expressed as percentage of WT maximal stimulation and represent the mean ± SEM of six independent experiments. **(B)** Time course of FSH-stimulated phosphorylation of ERK1/2 in cells expressing variants. Data are expressed as percentage of WT maximal stimulation and represent the mean ± SEM of three independent experiments. * *P* < 0.05, ** *P* < 0.01, *** *P* < 0.001 *vs.* WT *FSHR*. **(C)** Representative images of immunoblots using antibody against phosphorylated ERK1/2. Transfected cells were incubated with 500 IU FSH for the indicated time. **(D)** Bar plots showing quantitative fluorescence microscopy analysis of cell surface expression for all variants. Data are expressed as percentage of WT FSHR and represent the mean ± SEM of six independent experiments. *** P < 0.001 vs. WT *FSHR*, n.s. not significant. **(E)** Representative fluorescence microscopy images of the receptor cellular localization. Bar = 20 µm.

Examination of FSHR-mediated cAMP-dependent CRE-promoter transcription activity was conducted by co-transfection of HEK293 cells with the variants and CRE-luciferase reporter. In the presence of FSH, a dose-dependent increase in luciferase activity (EC_50_ = 11.98 IU) was recorded in cells harboring WT *FSHR* (**Figure 2A**). However, both FSH efficacy and potency to elicit cAMP-dependent CRE-reporter activity were significantly impaired in cells transfected with p.A621V variant as demonstrated by decreased maximal stimulation (59.72% of WT) and increased EC_50_ (81 IU) (**Figure 2A**). The most deteriorating impact in cAMP-dependent CRE-promoter activity was observed in cells expressing the p.A462P variant, whose luciferase activity was barely detectable (maximal stimulation = 4.24% of WT FSHR; EC_50_ = 32.3 IU) even in the presence 10,000 IU FSH, the highest concentration in our study (**Figure 2A**). When a blend of A462P and A621V was transfected together in equimolar concentration to mimic the compound heterozygous state found in the proband, there was a significant reduction either in the potency (EC_50_ = 62.16 IU) or in the efficacy (maximal stimulation = 44.95% of WT) as compared to those observed with the wild-type receptor.

### A462P and A621V variants were significantly impaired in ERK activation

To further explore whether ERK activation is altered by the variants, we performed Western immunoblot using a monoclonal antibody against phosphorylated ERK1/2. As shown in **Figures 2B, C**, FSHR-related ERK activation was time-dependent with a maximal activation at 5 min and a subsequent reduction to 42.14% of maximal levels at 10 min in WT receptor transfected HEK293 cells after stimulation with 500 IU FSH. We found that receptors homozygous for either variant exhibited significantly reduced ERK activation compared to the WT receptor (**Figures 2B, C**). The homozygous A462P variant receptor almost completely abolished ERK activation at both treatment periods, whereas homozygous A621V variant receptors carried 45.79% and 33.21% of maximal ERK activation at 5 and 10 min, respectively. When both A462P and A621V variants were expressed together to mimic the compound heterozygous state of the patient, the magnitude of ERK activation in response to 5 and 10 min of FSH stimulation was severely impaired compared with the WT FSHR (**Figures 2B, C**).

### Cell surface expression of the A462P variant was significantly impaired

Examination of the cell surface expression of p.A462P and p.A621V was conducted by transfection of HEK293 cells with these variants fused with EGFP. As shown in **Figures 2D, E**, the p.A621V variant and WT receptor exhibited comparable cell surface expression levels, but the p.A462P variant lost 50% of cell surface expression and was trapped intracellularly, suggesting that the nearly total loss of function in this variant might be due to reduced membrane receptor expression.

## Discussion

In this study, we investigated a case of primary infertility in a woman previously diagnosed with ovarian dysfunction who in fact had POI with ROS; we identified two novel missense variants in the *FSHR* gene, c.1384G>C/p.Ala462Pro and c.1862C>T/p.Ala621Val, inherited from her father and mother, respectively. These two variants were predicted to be disease causing by bioinformatics analysis. *In vitro* functional studies confirmed that these two variants are both pathogenic inactivating variants

FSHR variants are functionally distinct with respect to their ligand binding, membrane expression, cAMP production, and other intracellular signaling characteristics (17, 52). The complexity of these variants is further amplified by the fact that causative variant of POI can be either homozygous or compound heterozygous (19, 21, 30, 53, 54). As a consequence, in a single *FSHR* gene, genetic alteration can result in various clinical manifestations, leading to the broad phenotypic spectrum of conditions arising from these variants. Thus, correlation of novel *FSHR* variants with a new case of POI characterized by endogenous HH, normal ovarian reserve, and hypo-responsiveness to hyper-physiological exogenous GN stimulation testifies to the phenotypic and etiological complexity of POI.

Patients with partially inactivating *FSHR* variants have displayed a range of clinical manifestations such as primary amenorrhea, secondary amenorrhea, and oligomenorrhea, which is in contrast to completely inactivating *FSHR* variants that cause fully absence of sexual development (55). The patient in our study displayed normal puberty and secondary sex characteristics, and presented with secondary amenorrhea and normal-sized bilateral ovaries, in which developing follicles were detected by ultrasonography. This phenotype is notably differed from patients with severe variants (19, 36, 56). The term ROS has been used to describe this special form of POI, which is caused by FSH resistance rather than follicular depletion (21, 22, 26, 27, 33, 54). Although most ROS patients presented with developing antral follicles (2-8 mm) on ultrasonography and follicular arrest at the early antral stage on ovarian biopsies, further maturation of follicle is usually blocked in these patients (21, 22, 24–27). The patient’s follicles seemed incapable of proceeding through the later stages of follicular maturation, but likely produced adequate sex hormones at puberty to drive development of comparable secondary sex characteristics which accords with other studies with less severe variants (21, 22, 26, 27). The above surmise was supported by the normal AMH level detected in our case, which is consistent with previous studies that ROS patients have age-matched AMH values while patients with common POI have low or undetectable levels of AMH (25–27, 31, 54). These observations suggest that POI caused by inactivating *FSHR* variants could have wide-range effects on clinical manifestations, with disturbance severity likely varying with the severity of inactivation and the resultant stage of follicular block.

The FSHR protein is encoded by the *FSHR* gene positioned at chromosome 2p21 (57). The two variants herein are both positioned at exon 10, which is the largest exon, mainly responsible for encoding the non-ECD domains. The p.A462P and p.A621V variants are positioned at the TMH3 and TMH7 of the receptor, respectively, where no mutation had formerly been observed in patients. Our *in vitro* functional study demonstrated that the p.A462P variant causes the intracellular retention of the receptor, leading to impaired FSHR cell membrane expression. Several previous studies have reported the association of lower membrane expression of FSHR variants with impaired intracellular signaling of the receptor (19, 21, 22, 25, 29, 31, 54, 56). In our study, the lower membrane expression of the p.A462P variant was linked with an approximately 96% reduction in the maximal response elicited by the FSH. However, FSHR cell surface localization was not affected by the p.A621V variant, but its intracellular signaling was significantly impaired. Consistent with these results, three previously identified inactivating variants of *FSHR* showing intact cell surface expression displayed a dramatic reduction in cAMP signaling (21, 22, 27). Partially functional impaired FSHR caused by variants affects the further development of follicular growth (21, 28, 31, 55), indicating that the partial functional impairment of FSHR mediated by these two novel variants might explain the follicles advanced to the small antral phase and normal AMH levels found in this patient.

Even after receiving a maximal daily FSH dose (375 IU), the patient’s ovaries still remained unresponsive, which was consistent with several previous studies where partial or complete inactivating *FSHR* variants could not respond to intense and continual FSH stimulation (21, 22, 27, 28, 34, 36, 54, 58). It is worth noting that only one study has reported successful FSH treatment for ovarian stimulation in patient with ROS (24). The patient with successful treatment carried one heterozygous variant and two heterozygous SNPs. These clinical findings demonstrate that the differences in the ovarian responses to FSH stimulation noted between the successful and failed cases likely depend on the *FSHR* genotype. Patients who failed to react to follicular stimulation following an *in vitro* fertilization cycle could consider *in vitro* maturation (IVM) of the oocytes isolated from the blocked follicles. Successful IVM of oocytes was found in a primary infertility patient who carried compound heterozygous *FSHR* variants and had no response to FSH stimulation (54). Recently, Benammar et al. also reported a successful case of IVM live birth in a woman with primary infertility and ovarian resistance to FSH cause by compound heterozygous *FSHR* variants (58).

The lack of ovarian response to excessive FSH stimulation seems contradictory with the residual intracellular signaling observed *in vitro*. However, although *in vitro* functional data can be used as reference for *in vivo* scenarios, the selection of functional assay model may affect *in vitro* FSHR functionality (28). Moreover, receptor expression levels resulting from *in vitro* overexpression are far higher than in *in vivo* physiological environments, indicating that the real intracellular signaling of these variants in response to FSH stimulation would be probably poor *in vivo*. This observation suggests that in the presence of adequate FSH stimulation, partially functional but impaired FSHR might be critical for follicular growth.

In conclusion, we uncovered two novel *FSHR* variants, c.1384G>C/p.A462P and c.1862C>T/p.A621V, in a primary infertility patient affected by POI with ROS. To the best of our knowledge, these variants represent the first pathogenic inactivating variants found in the third and seventh TMH of FSHR. This study offers novel insights into the molecular basis of POI, expands the current knowledge of genotype–phenotype correlations and genetic variant spectrum of POI, and promotes the clinical diagnosis of patients with POI.

## Data availability statement

The datasets presented in this study can be found in online repositories. The names of the repository/repositories and accession number(s) can be found below: https://www.ncbi.nlm.nih.gov/, PRJNA687912; https://www.ncbi.nlm.nih.gov/, SCV001478347; https://www.ncbi.nlm.nih.gov/, SCV001478348.

## Ethics statement

The studies involving human participants were reviewed and approved by The Institutional Ethics Committee of Zhejiang provincial People’ Hospital. The patients/participants provided their written informed consent to participate in this study.

## Author contributions

XC was responsible for the experimental design, critical data analysis, interpretation, article writing and editing, and participated in conducting experimental procedures and coordination. LC, YW, CS, YZ, and LY conducted the main experimental procedures, and participated in experimental protocol design, data analysis and interpretation. BJ and RW contributed to the clinical data collection and interpretation. JHS and MQ contributed to the genetic counseling and data interpretation. JS conceived the study, participated in experimental design and coordination, and was responsible for the project supervision and final approval of the article. All authors contributed to the article and approved the submission version.

## References

[1] Nelson LM. Clinical practice. primary ovarian insufficiency. N Engl J Med (2009) 360(6):606–14. doi: 10.1056/NEJMcp0808697 PMC2762081 19196677

[2] Welt CK. Primary ovarian insufficiency: A more accurate term for premature ovarian failure. Clin Endocrinol (Oxf) (2008) 68(4):499–509. doi: 10.1111/j.1365-2265.2007.03073.x 17970776

[3] Coulam CB Adamson SC Annegers JF. Incidence of premature ovarian failure. Obstet Gynecol (1986) 67(4):604–6. 3960433

[4] Jones GS De Moraes-Ruehsen M. A new syndrome of amenorrhae in association with hypergonadotropism and apparently normal ovarian follicular apparatus. Am J Obstet Gynecol (1969) 104(4):597–600. doi: 10.1016/S0002-9378(16)34255-7 5786709

[5] Koninckx PR Brosens IA. The "gonadotropin-resistant ovary" syndrome as a cause of secondary amenorrhea and infertility. Fertil Steril (1977) 28(9):926–31. doi: 10.1016/S0015-0282(16)42792-5 892043

[6] Shangold MM Turksoy RN Bashford RA Hammond CB. Pregnancy following the "insensitive ovary syndrome". Fertil Steril (1977) 28(11):1179–81. doi: 10.1016/S0015-0282(16)42914-6 923834

[7] Huhtaniemi I Hovatta O La Marca A Livera G Monniaux D Persani L. Advances in the molecular pathophysiology, genetics, and treatment of primary ovarian insufficiency. Trends Endocrinol Metab (2018) 29(6):400–19. doi: 10.1016/j.tem.2018.03.010 29706485

[8] Qin Y Jiao X Simpson JL Chen ZJ. Genetics of primary ovarian insufficiency: new developments and opportunities. Hum Reprod Update (2015) 21(6):787–808. doi: 10.1093/humupd/dmv036 26243799 PMC4594617

[9] Jaillard S Bell K Akloul L Walton K McElreavy K Stocker WA. New insights into the genetic basis of premature ovarian insufficiency: Novel causative variants and candidate genes revealed by genomic sequencing. Maturitas (2020) 141:9–19. doi: 10.1016/j.maturitas.2020.06.004 33036707

[10] Oktay K Briggs D Gosden RG. Ontogeny of follicle-stimulating hormone receptor gene expression in isolated human ovarian follicles. J Clin Endocrinol Metab (1997) 82(11):3748–51. doi: 10.1210/jc.82.11.3748 9360535

[11] Simoni M Gromoll J Nieschlag E. The follicle-stimulating hormone receptor: Biochemistry, molecular biology, physiology, and pathophysiology. Endocr Rev (1997) 18(6):739–73. doi: 10.1210/edrv.18.6.0320 9408742

[12] Richards JS Pangas SA. The ovary: Basic biology and clinical implications. J Clin Invest (2010) 120(4):963–72. doi: 10.1172/JCI41350 PMC2846061 20364094

[13] Jiang X Liu H Chen X Chen PH Fischer D Sriraman V. Structure of follicle-stimulating hormone in complex with the entire ectodomain of its receptor. Proc Natl Acad Sci USA (2012) 109(31):12491–6. doi: 10.1073/pnas.1206643109 PMC3411987 22802634

[14] Fredriksson R Lagerstrom MC Lundin LG Schioth HB. The G-protein-coupled receptors in the human genome form five main families. phylogenetic analysis, paralogon groups, and fingerprints. Mol Pharmacol (2003) 63(6):1256–72. doi: 10.1124/mol.63.6.1256 12761335

[15] Jiang X Dias JA He X. Structural biology of glycoprotein hormones and their receptors: Insights to signaling. Mol Cell Endocrinol (2014) 382(1):424–51. doi: 10.1016/j.mce.2013.08.021 24001578

[16] Casarini L Crepieux P. Molecular mechanisms of action of FSH. Front Endocrinol (Lausanne) (2019) 10:305. doi: 10.3389/fendo.2019.00305 31139153 PMC6527893

[17] Ulloa-Aguirre A Zarinan T Jardon-Valadez E Gutierrez-Sagal R Dias JA. Structure-function relationships of the follicle-stimulating hormone receptor. Front Endocrinol (Lausanne) (2018) 9:707. doi: 10.3389/fendo.2018.00707 30555414 PMC6281744

[18] Desai SS Roy BS Mahale SD. Mutations and polymorphisms in FSH receptor: functional implications in human reproduction. Reproduction (2013) 146(6):R235–48. doi: 10.1530/REP-13-0351 24051057

[19] Aittomaki K Lucena JL Pakarinen P Sistonen P Tapanainen J Gromoll J. Mutation in the follicle-stimulating hormone receptor gene causes hereditary hypergonadotropic ovarian failure. Cell (1995) 82(6):959–68. doi: 10.1016/0092-8674(95)90275-9 7553856

[20] Gromoll J Simoni M Nordhoff V Behre HM De Geyter C Nieschlag E. Functional and clinical consequences of mutations in the FSH receptor. Mol Cell Endocrinol (1996) 125(1-2):177–82. doi: 10.1016/S0303-7207(96)03949-4 9027356

[21] Beau I Touraine P Meduri G Gougeon A Desroches A Matuchansky C. A novel phenotype related to partial loss of function mutations of the follicle stimulating hormone receptor. J Clin Invest (1998) 102(7):1352–9. doi: 10.1172/JCI3795 PMC508982 9769327

[22] Touraine P Beau I Gougeon A Meduri G Desroches A Pichard C. New natural inactivating mutations of the follicle-stimulating hormone receptor: Correlations between receptor function and phenotype. Mol Endocrinol (1999) 13(11):1844–54. doi: 10.1210/mend.13.11.0370 10551778

[23] Allen LA Achermann JC Pakarinen P Kotlar TJ Huhtaniemi IT Jameson JL. A novel loss of function mutation in exon 10 of the FSH receptor gene causing hypergonadotrophic hypogonadism: Clinical and molecular characteristics. Hum Reprod (2003) 18(2):251–6. doi: 10.1093/humrep/deg046 12571157

[24] Nakamura Y Maekawa R Yamagata Y Tamura I Sugino N. A novel mutation in exon8 of the follicle-stimulating hormone receptor in a woman with primary amenorrhea. Gynecol Endocrinol (2008) 24(12):708–12. doi: 10.1080/09513590802454927 19172541

[25] Liu H Xu X Han T Yan L Cheng L Qin Y. A novel homozygous mutation in the FSHR gene is causative for primary ovarian insufficiency. Fertil Steril (2017) 108(6):1050–5 e2. doi: 10.1016/j.fertnstert.2017.09.010 29157895

[26] He WB Du J Yang XW Li W Tang WL Dai C. Novel inactivating mutations in the FSH receptor cause premature ovarian insufficiency with resistant ovary syndrome. Reprod BioMed Online (2019) 38(3):397–406. doi: 10.1016/j.rbmo.2018.11.011 30691934

[27] Khor S Lyu Q Kuang Y Lu X. Novel FSHR variants causing female resistant ovary syndrome. Mol Genet Genomic Med (2020) 8(2):e1082. doi: 10.1002/mgg3.1082 31830376 PMC7005632

[28] Sassi A Désir J Janssens Véronique Marangoni M Daneels D Gheldof A. Novel inactivating follicle-stimulating hormone receptor (FSHR) mutations in a patient with premature ovarian insufficiency identified by next generation sequencing gene panel analysis. F&S Rep (2020) 1(3):193–201. doi: 10.1016/j.xfre.2020.08.008 PMC8244262 34223243

[29] Liu H Guo T Gong Z Yu Y Zhang Y Zhao S. Novel FSHR mutations in han Chinese women with sporadic premature ovarian insufficiency. Mol Cell Endocrinol (2019) 492:110446. doi: 10.1016/j.mce.2019.05.005 31077743

[30] Doherty E Pakarinen P Tiitinen A Kiilavuori A Huhtaniemi I Forrest S. A novel mutation in the FSH receptor inhibiting signal transduction and causing primary ovarian failure. J Clin Endocrinol Metab (2002) 87(3):1151–5. doi: 10.1210/jcem.87.3.8319 11889179

[31] Bramble MS Goldstein EH Lipson A Ngun T Eskin A Gosschalk JE. A novel follicle-stimulating hormone receptor mutation causing primary ovarian failure: A fertility application of whole exome sequencing. Hum Reprod (2016) 31(4):905–14. doi: 10.1093/humrep/dew025 PMC5007606 26911863

[32] Katari S Wood-Trageser MA Jiang H Kalynchuk E Muzumdar R Yatsenko SA. Novel inactivating mutation of the FSH receptor in two siblings of Indian origin with premature ovarian failure. J Clin Endocrinol Metab (2015) 100(6):2154–7. doi: 10.1210/jc.2015-1401 PMC5393517 25875778

[33] Kuechler A Hauffa BP Koninger A Kleinau G Albrecht B Horsthemke B. An unbalanced translocation unmasks a recessive mutation in the follicle-stimulating hormone receptor (FSHR) gene and causes FSH resistance. Eur J Hum Genet (2010) 18(6):656–61. doi: 10.1038/ejhg.2009.244 PMC2987335 20087398

[34] Zarinan T Mayorga J Jardon-Valadez E Gutierrez-Sagal R Maravillas-Montero JL Mejia-Dominguez NR. A novel mutation in the FSH receptor (I423T) affecting receptor activation and leading to primary ovarian failure. J Clin Endocrinol Metab (2021) 106(2):e534–e50. doi: 10.1210/clinem/dgaa782 33119067

[35] Franca MM Lerario AM Funari MFA Nishi MY Narcizo AM de Mello MP. A novel homozygous missense FSHR variant associated with hypergonadotropic hypogonadism in two siblings from a Brazilian family. Sex Dev (2017) 11(3):137–42. doi: 10.1159/000477193 28591755

[36] Meduri G Touraine P Beau I Lahuna O Desroches A Vacher-Lavenu MC. Delayed puberty and primary amenorrhea associated with a novel mutation of the human follicle-stimulating hormone receptor: Clinical, histological, and molecular studies. J Clin Endocrinol Metab (2003) 88(8):3491–8. doi: 10.1210/jc.2003-030217 12915623

[37] Woad KJ Prendergast D Winship IM Shelling AN. FSH receptor gene variants are rarely associated with premature ovarian failure. Reprod BioMed Online (2013) 26(4):396–9. doi: 10.1016/j.rbmo.2013.01.004 23419799

[38] European Society for Human R Embryology Guideline Group on POI Webber L Davies M Anderson R Bartlett J. ESHRE guideline: Management of women with premature ovarian insufficiency. Hum Reprod (2016) 31(5):926–37. doi: 10.1093/humrep/dew027 27008889

[39] Genomes Project C Auton A Brooks LD Durbin RM Garrison EP Kang HM. A global reference for human genetic variation. Nature (2015) 526(7571):68–74. doi: 10.1038/nature15393 26432245 PMC4750478

[40] Karczewski KJ Francioli LC Tiao G Cummings BB Alfoldi J Wang Q. The mutational constraint spectrum quantified from variation in 141,456 humans. Nature (2020) 581(7809):434–43. doi: 10.1038/s41586-020-2308-7 PMC7334197 32461654

[41] Sherry ST Ward MH Kholodov M Baker J Phan L Smigielski EM. dbSNP: The NCBI database of genetic variation. Nucleic Acids Res (2001) 29(1):308–11. doi: 10.1093/nar/29.1.308 PMC29783 11125122

[42] Amberger JS Bocchini CA Schiettecatte F Scott AF Hamosh A. OMIM.org: Online mendelian inheritance in man (OMIM(R)), an online catalog of human genes and genetic disorders. Nucleic Acids Res (2015) 43(Database issue):D789–98. doi: 10.1093/nar/gku1205 PMC4383985 25428349

[43] Landrum MJ Lee JM Riley GR Jang W Rubinstein WS Church DM. ClinVar: public archive of relationships among sequence variation and human phenotype. Nucleic Acids Res (2014) 42(Database issue):D980–5. doi: 10.1093/nar/gkt1113 PMC3965032 24234437

[44] Fokkema IF Taschner PE Schaafsma GC Celli J Laros JF den Dunnen JT. LOVD v.2.0: The next generation in gene variant databases. Hum Mutat (2011) 32(5):557–63. doi: 10.1002/humu.21438 21520333

[45] Richards S Aziz N Bale S Bick D Das S Gastier-Foster J. Standards and guidelines for the interpretation of sequence variants: A joint consensus recommendation of the American college of medical genetics and genomics and the association for molecular pathology. Genet Med (2015) 17(5):405–24. doi: 10.1038/gim.2015.30 PMC4544753 25741868

[46] Krogh A Larsson B von Heijne G Sonnhammer EL. Predicting transmembrane protein topology with a hidden Markov model: Application to complete genomes. J Mol Biol (2001) 305(3):567–80. doi: 10.1006/jmbi.2000.4315 11152613

[47] Adzhubei IA Schmidt S Peshkin L Ramensky VE Gerasimova A Bork P. A method and server for predicting damaging missense mutations. Nat Methods (2010) 7(4):248–9. doi: 10.1038/nmeth0410-248 PMC2855889 20354512

[48] Ng PC Henikoff S. SIFT: Predicting amino acid changes that affect protein function. Nucleic Acids Res (2003) 31(13):3812–4. doi: 10.1093/nar/gkg509 PMC168916 12824425

[49] Schwarz JM Cooper DN Schuelke M Seelow D. MutationTaster2: mutation prediction for the deep-sequencing age. Nat Methods (2014) 11(4):361–2. doi: 10.1038/nmeth.2890 24681721

[50] Zhu C Huang H Hua R Li G Yang D Luo J. Molecular and functional characterization of adipokinetic hormone receptor and its peptide ligands in bombyx mori. FEBS Lett (2009) 583(9):1463–8. doi: 10.1016/j.febslet.2009.03.060 PMC2745557 19345219

[51] Chen XP Yang W Fan Y Luo JS Hong K Wang Z. Structural determinants in the second intracellular loop of the human cannabinoid CB1 receptor mediate selective coupling to g(s) and g(i). Br J Pharmacol (2010) 161(8):1817–34. doi: 10.1111/j.1476-5381.2010.01006.x PMC3010585 20735408

[52] Ulloa-Aguirre A Reiter E Crepieux P. FSH receptor signaling: Complexity of interactions and signal diversity. Endocrinology (2018) 159(8):3020–35. doi: 10.1210/en.2018-00452 29982321

[53] Latronico AC Arnhold IJ. Gonadotropin resistance. Endocr Dev (2013) 24:25–32. doi: 10.1159/000342496 23392092

[54] Flageole C Toufaily C Bernard DJ Ates S Blais V Chenier S. Successful *in vitro* maturation of oocytes in a woman with gonadotropin-resistant ovary syndrome associated with a novel combination of FSH receptor gene variants: A case report. J Assist Reprod Genet (2019) 36(3):425–32. doi: 10.1007/s10815-018-1394-z PMC6439039 30610662

[55] Oduwole OO Huhtaniemi IT Misrahi M. The roles of luteinizing hormone, follicle-stimulating hormone and testosterone in spermatogenesis and folliculogenesis revisited. Int J Mol Sci (2021) 22(23):12735. doi: 10.3390/ijms222312735 34884539 PMC8658012

[56] Desai SS Achrekar SK Sahasrabuddhe KA Meharji PK Desai SK Mangoli VS. Functional characterization of two naturally occurring mutations (Val514Ala and Ala575Val) in follicle-stimulating hormone receptor. J Clin Endocrinol Metab (2015) 100(4):E638–45. doi: 10.1210/jc.2014-3662 25581598

[57] Gromoll J Pekel E Nieschlag E. The structure and organization of the human follicle-stimulating hormone receptor (FSHR) gene. Genomics (1996) 35(2):308–11. doi: 10.1006/geno.1996.0361 8661143

[58] Benammar A Fanchin R Filali-Baba M Vialard F Fossard C Vandame J. Utilization of *in vitro* maturation in cases with a FSH receptor mutation. J Assist Reprod Genet (2021) 38(6):1311–21. doi: 10.1007/s10815-021-02249-3 PMC8266928 34089127

