# Supplementary figures and images for: Identification and characterization of novel compound heterozygous variants in FSHR causing primary ovarian insufficiency with resistant ovary syndrome
Source: Front Endocrinol (Lausanne). 2023 Jan 10;13:1013894. doi: 10.3389/fendo.2022.1013894 (PMC9871476; doi:10.3389/fendo.2022.1013894)

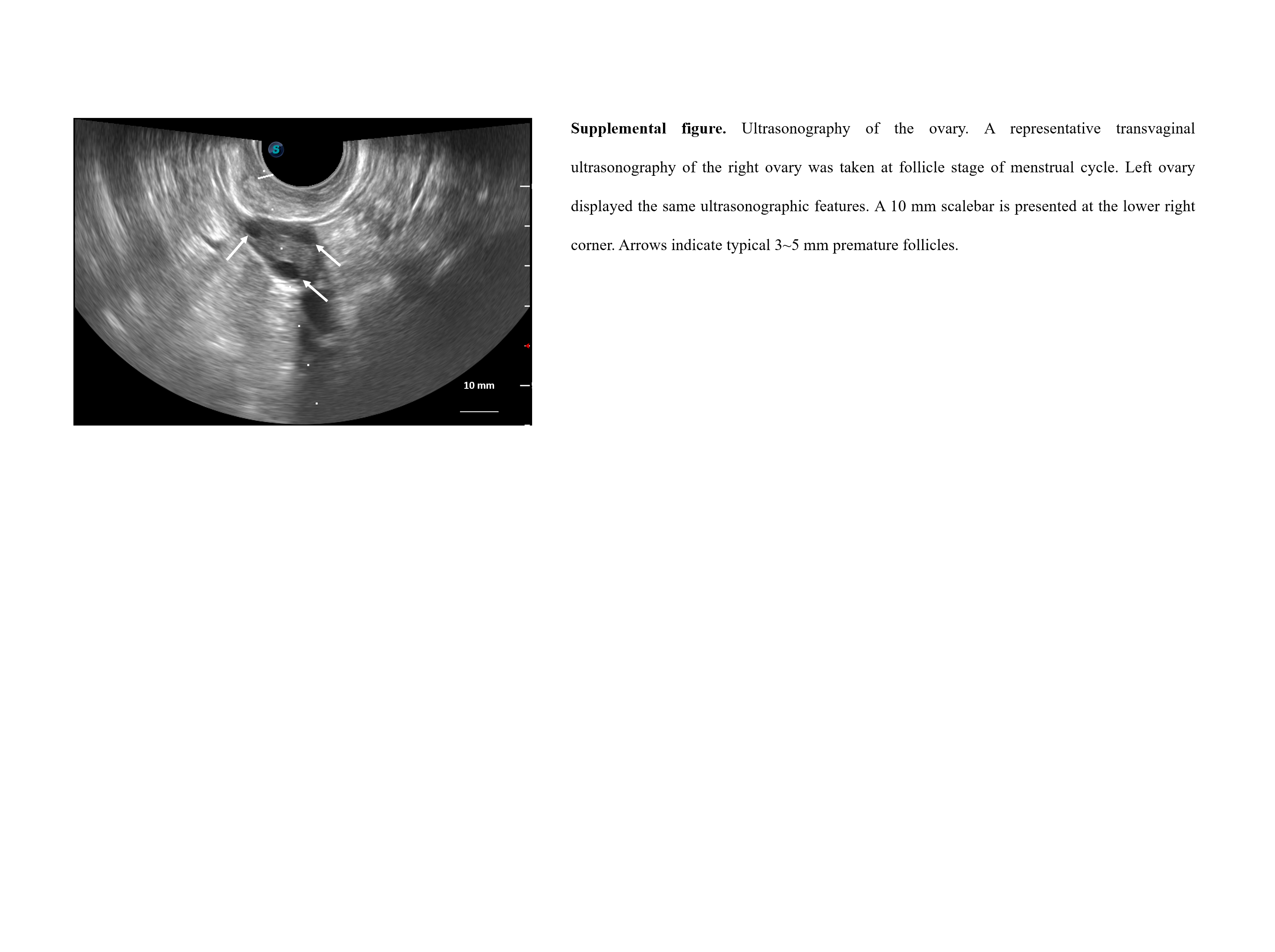

Supplement: Supplementary file 1 [file Image_1.tif]

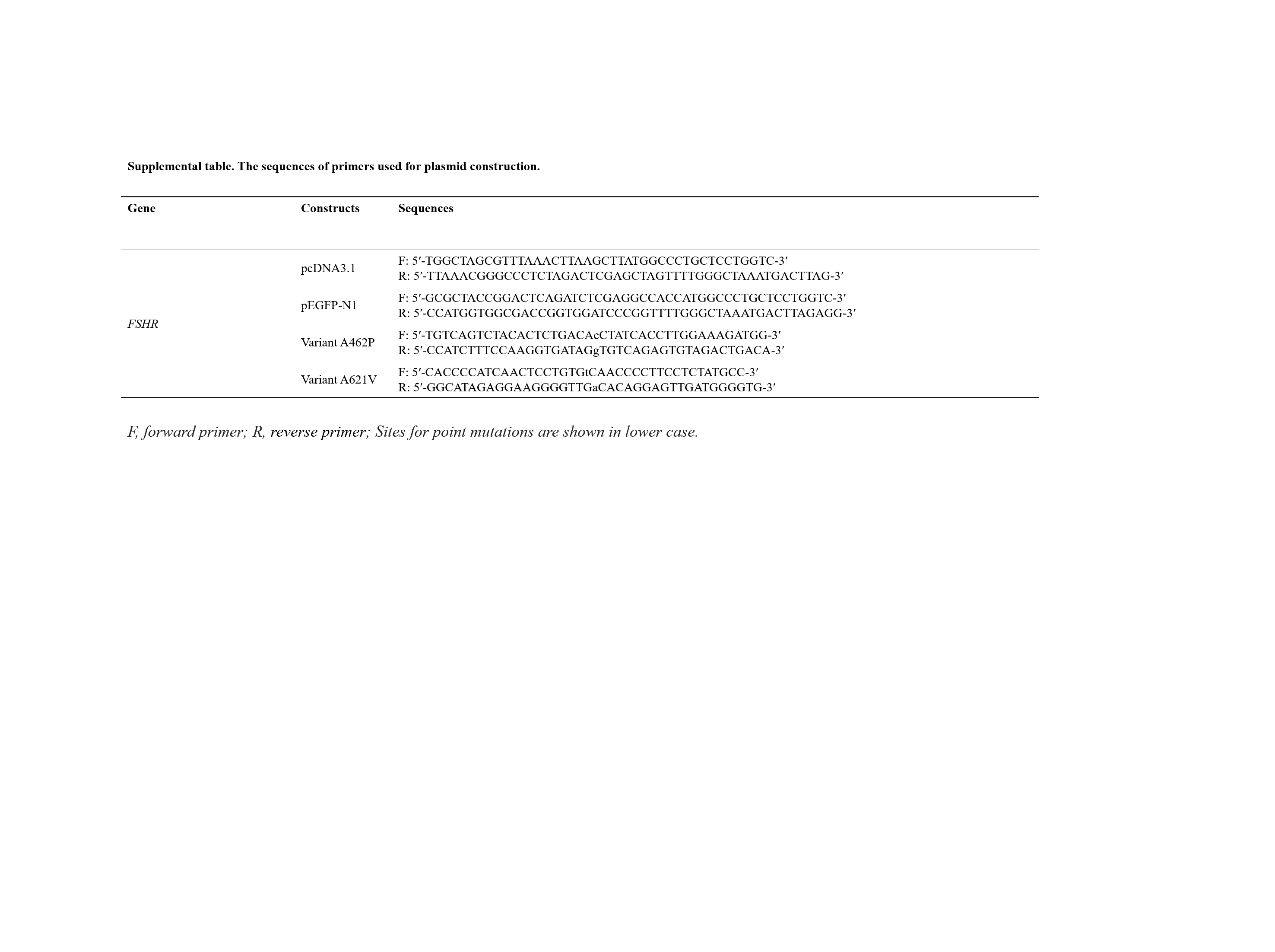

Supplement: Supplementary file 2 [file Image_2.tif]
